# Circadian rhythm, glucose metabolism and diabetic complications: the role of glucokinase and the enlightenment on future treatment

**DOI:** 10.3389/fphys.2025.1537231

**Published:** 2025-02-21

**Authors:** Zhijun Zhang, Shuo Wang, Ling Gao

**Affiliations:** Department of Endocrinology and Metabolism, Renmin Hospital of Wuhan University, Wuhan, China

**Keywords:** circadian rhythm, disruption, glucose metabolism, diabetes, diabetic complications, glucokinase

## Abstract

The circadian clock, an innate timing mechanism, governs a variety of physiological activities by producing near-24-h cycles in gene expression. These cycles are reflected in patterns of metabolism and behavior. This system consists of two parts: one is the central clock located in the suprachiasmatic nucleus of the hypothalamus, and the other is the peripheral clock located in tissues throughout the body. Glucokinase, also termed hexokinase 4, is a member of the hexokinase family. It acts as a glucose sensor, plays a pivotal role in glucose homeostasis. Here, we review the role of circadian rhythm in glucose metabolism across various tissues, look into the molecular mechanism of circadian disruption involvement in glucose metabolism and diabetic complications, with a particular focus on the role of glucokinase. Finally, we propose potential strategies for effectively treating metabolic disorders and diabetic complications by modulating circadian rhythm glucokinase.

## 1 Introduction

Disruption of circadian rhythms is a frequently overlooked risk factor for diabetes (Stenvers et al., 2019). The circadian system, often referred to as the biological clock. It consists of two parts: one is the central clock located in the suprachiasmatic nucleus of the hypothalamus, and the other is the peripheral clock located in tissues throughout the body. These clocks are synchronized by neuronal and hormonal signals, body temperature, light, and feeding cues (Reppert and Weaver, 2002). Increasing evidence suggests that various clock genes play roles in lipid regulation, glucose balance and overall health (Manoogian and Panda, 2017; Poggiogalle et al., 2018). Recent research indicates a strong link between circadian disruption and the development of diabetes and its complications (Potter et al., 2016; Mason et al., 2020; Nakazawa et al., 2022).

Glucokinase (GCK), also known as hexokinase 4, belongs to the hexokinase family and acts as a glucose sensor pivotal for glucose homeostasis. While other hexokinases (HK1–3) exhibit high affinity for glucose (saturated at fasting levels of ∼5 mM) and are inhibited by glucose-6-phosphate (G6P), GCK has a low affinity for glucose (EC50 ∼8–10 mM), is not inhibited by G6P, and phosphorylates glucose proportionally across a broader physiological range (3–15 mM) (Ashcroft et al., 2023). These unique characteristics enable GCK to dynamically regulate glucose utilization and storage in response to postprandial glycemic fluctuations, making it a critical player in metabolic regulation and a key target for studying circadian rhythms in glucose metabolism.

The expression and activity of glucokinase are influenced by circadian rhythms, linking it directly to the metabolic disturbances observed in diabetes Studies have shown that the expression and activity of GCK exhibit circadian rhythmic fluctuations. Typically, GCK activity is increased after feeding but decreased during fasting, which helps process glucose absorbed from food. This is closely linked to the feeding-fasting cycle in animals. The circadian expression of GCK is directly controlled by the circadian locomotor output cycles kaput (CLOCK) and aryl hydrocarbon receptor nuclear translocator-like protein 1 (BMAL1), which bind to the E-box at the promoter region of GCK, making it a clock-controlled gene (CCG). Disruptions in these circadian genes may lead to abnormal GCK function, thereby affecting glucose metabolism (Llanos et al., 2023b). In addition to circadian regulation, GCK expression is also modulated by dietary signals. GCK is regulated by dietary signals primarily through the insulin-mediated sterol regulatory element-binding protein 1c (SREBP1c) pathway and direct glucose regulation. Postprandial insulin activates SREBP1c, enhancing GCK transcription, while elevated glucose levels directly upregulate GCK expression (Kim et al., 2004). Additionally, pathways such as Glucagon-like peptide-1 (GLP-1) and Liver X Receptor (LXR) further modulate GCK, enabling it to dynamically respond to dietary changes and maintain glucose homeostasis (Kim et al., 2009; Ding et al., 2011b).

In recent years, GCK activators have been considered an effective antidiabetic drug. However, the impact of GCK on lipid metabolism should not be overlooked. Activation of GCK leads to hepatic lipid accumulation and inflammation by altering the expression of liver genes involved in lipogenesis, lipolysis, and β-oxidation (Xie et al., 2023). Therefore, GCK activators can contradictorily affect both glucose and lipid metabolism, sometimes improving glucose control while potentially disrupting lipid balance (Jiang et al., 2024). This paradox underscores the complexity of targeting GCK for therapeutic purposes and highlights the importance of considering circadian rhythms in the development of treatments targeting GCK and metabolic disorders.

In this review, we will introduce the concept of circadian rhythms, analyze the impact of circadian rhythm disruption on diabetes and its complication, with a particular focus on the involvement of glucokinase. We will look into the molecular mechanism of circadian rhythm affecting these diseases, and explore the promising treatment strategies for diabetes and its complication.

## 2 General mechanisms of circadian rhythm

The circadian clock is an internal system that predicts daily environmental changes (Young and Kay, 2001). In mammals, a central clock is located in the hypothalamic suprachiasmatic nuclei (SCN) and primarily responds to the light-dark cycle (Hastings et al., 2018). Peripheral clocks, similar to the SCN, exist in tissues such as the liver and pancreas (Mohawk et al., 2012). The circadian clock in every cell relies on a transcriptional–translational feedback loop composed of clock genes. Core mammalian clock genes include CLOCK and BMAL1, which form BMAL1/CLOCK heterodimers. The first loop involves cryptochrome (CRY) and period (PER) proteins. During the rest phase, the BMAL1/CLOCK heterodimer binds to E-Box DNA binding sequences (DBS), promoting the expression of CRY and PER. These proteins then form dimers, move into the nucleus, and inhibit BMAL1/CLOCK activity during the active phase. Post-translational modifications lead to the degradation of PER and CRY, initiating the next circadian cycle. In the rest phase, REV-ERB represses BMAL1 and CLOCK, while during the active phase, decreasing REV-ERB and increasing ROR levels activate their expression, maintaining the circadian rhythm (Asher and Schibler, 2011; Bass, 2012; Takahashi, 2017). Overall, circadian clock possesses intricate molecular mechanism and is present in various tissues and organs throughout the body (Ardlie et al., 2015; Mure et al., 2018).

## 3 Circadian rhythms and glucose metabolism: the role of GCK

**FIGURE 1:**
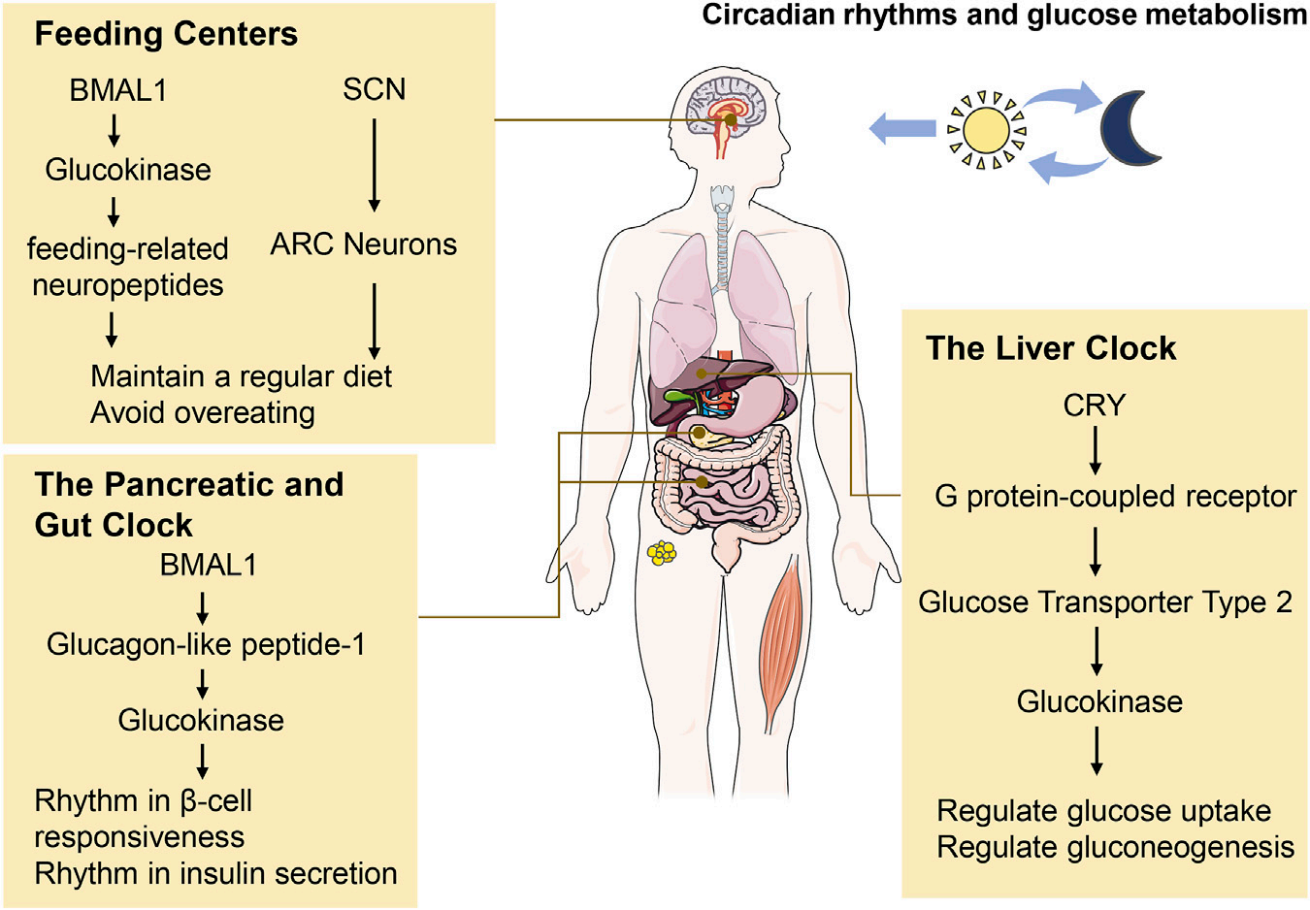
Circadian clocks regulate glucose metabolism. Circadian rhythms govern a large array of physiological and metabolic functions and permeate all levels of mammalian organization, especially in glucose metabolism. The main involvement of GCK is in the regulation of food intake and the control of peripheral tissue clocks, including the liver, pancreas, and gut. BMAL1, aryl hydrocarbon receptor nuclear translocator-like protein 1; CRY, cryptochrome; SCN, suprachiasmatic nuclei; ARC, arcuate nucleus.

Circadian rhythms regulate numerous physiological and metabolic functions, notably influencing glucose metabolism across all levels of mammalian organization. In healthy humans, plasma glucose concentrations are controlled at stable levels (Aparicio et al., 1974). Numerous human studies have documented a circadian rhythm in oral glucose tolerance, which usually peaks in the morning and declines in the afternoon and evening (Carroll and Nestel, 1973; Mayer et al., 1976; Wojtczak-Jaroszowa, 1977). As mentioned above, GCK activity is higher during feeding periods (typically during the day) and lower during fasting periods (typically at night). Therefore, the circadian rhythm of glucose tolerance may result from the combined actions of various peripheral tissues and GCK (Figure 1).

### 3.1 The pancreatic and gut clock

Insulin secretion exhibits a circadian rhythm. Insulin secretion rate peaks in the mid-afternoon and is lowest at night while sleeping. The rhythm may be associated with habitual feeding times, consistent with GCK (Boden et al., 1996; Saad et al., 2012). Circadian clock genes are involved in various pathways for insulin secretion. GLP-1, an incretin hormone primarily produced by intestinal L-cells, is also synthesized by pancreatic α-cells. It plays a vital role in insulin secretion (Seino et al., 2016). Research indicates that GLP-1 stimulates insulin secretion *via* a mechanism involving glucokinase (GCK) and is regulated by the clock gene BMAL1 (Ding et al., 2011a; Biancolin et al., 2020). Therefore, in β-cells, clock genes may affect the rhythm of insulin secretion through the GLP-1-GCK pathway.

### 3.2 The liver clock

The liver’s circadian clocks employ various mechanisms to produce antiphasic rhythms in glucose metabolism. During habitual feeding periods, the liver clock gene CRY plays an important role in metabolic regulation by modulating the expression of Glucose transporter type 2 (GLUT2). CRY achieves this by inhibiting G protein-coupled receptor (GPCR) signaling pathways, which otherwise would downregulate GLUT2 expression. The upregulation of GLUT2 enhances the activity of Glucokinase (GCK), a key enzyme in glucose metabolism. This activation of GCK facilitates hepatic glucose uptake, thereby reducing blood glucose levels, and concurrently suppresses hepatic gluconeogenesis, the process by which the liver produces glucose (Kinsella et al., 2021). The overall result produces nearly constant blood levels of glucose throughout the day (Zhang et al., 2010; Lamia et al., 2011).

### 3.3 The clocks of other tissues

The circadian rhythm of glucose metabolism involves not only the pancreas and liver mentioned above, but also other peripheral tissues suck as the adipose tissue, kidneys, and muscles. These tissues are regulated by BMAL1, PER1, and CRY. This regulation causes a circadian rhythm in insulin sensitivity in muscle and adipose tissue, and glucose reabsorption in the kidney, thereby influencing the overall circadian rhythm of glucose metabolism (Verrillo et al., 1989; Iwashina et al., 2011; Barclay et al., 2013; Solocinski et al., 2015; Nikolaeva et al., 2016; Gliniak et al., 2017; Ansermet et al., 2022). However, the expression of GCK in these tissues is minimal. Hexokinase 2 (HK2) is another isoform of HKs, present in almost all tissues. Circadian rhythms may influence insulin sensitivity in muscle and adipose tissue through the BMAL1-HK2-glucose transporter (Glut4) pathway (Harfmann et al., 2016; Shimobayashi et al., 2023). HK2 is also associated with glycogen deposition in the kidneys. Therefore, the regulation of renal glucose reabsorption by circadian rhythms may also involve HK2 (Rabbani et al., 2022).

## 4 Circadian rhythms on hypothalamic feeding centers -food intake

Researches find that the occurrence of glucose metabolic rhythm may be related to food intake (Zhao et al., 2021). This impact is mainly manifested in two aspects (Figure 1). On the one hand, there is a direct neuroanatomical connection between the SCN and the hypothalamic arcuate nucleus (ARC), the center for regulating food intake. The neuronal activity of ARC increases during hypoglycemia. SCN inhibits the activity of ARC neurons, avoiding the occurrence of more food intake and higher blood sugar levels after hypoglycemia (Herrera-Moro Chao et al., 2016). On the other hand, feeding-related neuropeptides expressed in the ARC are also affected by circadian clock genes, including the potent orexigenic hypothalamic neuropeptides, neuropeptide Y (NPY) and agouti-related peptide (AgRP), and the anorexigenic peptide α-melanocyte-stimulating hormone (POMC). The expression of these neuropeptides peak during eating. BMAL1 can inhibit the high expression of AgRP, NPY, and POMC, thereby avoiding overeating during both day and night (Clemenzi et al., 2020). Interestingly, GCK is widely expressed in the central nervous system, particularly in ARC of the hypothalamus. Previous studies have confirmed that GCK regulates AgRP and NPY in the hypothalamus. Since GCK is also influenced by circadian clock genes, the circadian rhythm of food intake might be related to GCK (Maria Uranga et al., 2017).

## 5 Role of circadian disruption in diabetic complications

Circadian disruption will increase the susceptibility to diabetes complications. A case-control study shows that service workers, who have sometimes to work in shifts and eat at irregular times, are more likely to have complications of diabetes (Nakazawa et al., 2022).

**FIGURE 2:**
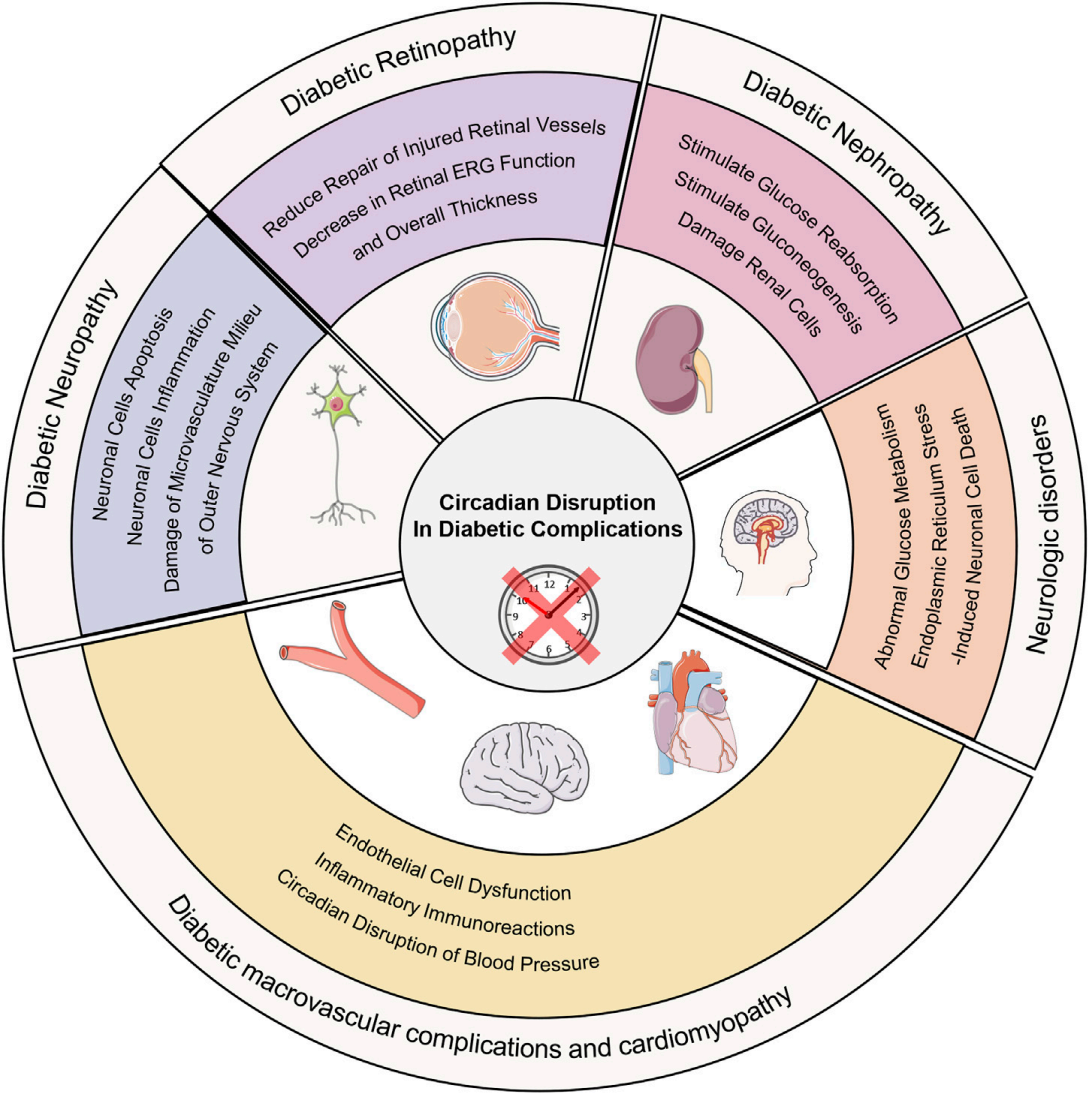
Circadian disruption in diabetic complications. Circadian disruption increases the occurrence of diabetic complications and its possible molecular mechanism.

In the following discussion we emphasize the epidemiological and background evidence linking circadian disruption to diabetic complications, and then explore examples of targetable mechanisms involving circadian disruption and GCK in these conditions (Figure 2).

### 5.1 Diabetic neuropathy

Diabetic neuropathy, affecting over 50% of those with diabetes, involves peripheral and autonomic nervous system damage, resulting in pain, increased fall risk, and reduced quality of life (Feldman et al., 2019). Circadian disruption may cause imbalance between free radicals’ production and clearance, hyperactivation of ERK-MAPK signaling pathway and abnormal expression of proinflammatory factors leading to damage of microvasculature milieu of outer nervous system, neuronal cells inflammation and neuronal cells apoptosis. They aggravate the occurrence and development of diabetic neuropathy (Daulhac et al., 2006; Patel et al., 2009; Prather et al., 2009; Zielinski et al., 2016; Griffin et al., 2019; Fang et al., 2021; Budkowska et al., 2022). TNF-α is an inflammatory protein upregulated by various mediators. Research has shown that activation of GCK activity can result in elevated TNF-α levels. TNF-α may induce inflammation and neuronal damage in the stellate ganglion, impairing its ability to regulate the heart (Xu et al., 2020). The expression level of GCK is influenced by BMAL1 (Llanos et al., 2023a). Therefore, circadian disruption may lead to the development of diabetic autonomic neuropathy through this inflammatory mechanism.

### 5.2 Diabetic macrovascular complications and cardiomyopathy

Macrovascular complications of diabetes mellitus, including cardiovascular diseases, peripheral artery disease and cerebrovascular disease, and cardiomyopathy are the primary causes of mortality in diabetic patients. Evidence is mounting which links circadian rhythm and its effects to macrovascular complications and cardiomyopathy of diabetes. Humans those who suffer from sleep disorders and animal with environmental circadian disruption may have an increased chance of developing macrovascular complications and cardiomyopathy (Baguet et al., 2012; Earnest et al., 2016; Kecklund and Axelsson, 2016; Pan et al., 2016; Qiao et al., 2017; Yang et al., 2023). The influence of circadian rhythm on those may be multifaceted including circadian disruption of blood pressure, inflammation, vascular endothelial dysfunction, and so on (Stamler et al., 1993; Gibbs et al., 2014; Kokubo and Iwashima, 2015; Frati et al., 2017; Nishida and Otsu, 2017; Gonzalez-Guerra et al., 2021; Kelly et al., 2021; Kong et al., 2022). Current research has found that the lack of GCK accelerates the development of atherosclerosis and cardiomyopathy (Li et al., 2014; Adingupu et al., 2016). Activation of GCK can reduce the risk of cardiovascular disease in diabetes (Wang et al., 2022). GCK may be involved in circadian disruption leading to diabetic macrovascular diseases, but its specific mechanisms require further clarification.

### 5.3 Diabetic nephropathy (DN) and diabetic retinopathy (DR)

Recent research has suggested that diabetic nephropathy and retinopathy will occur and progress more quickly due to circadian disruption (Firsov and Bonny, 2018). Disturbances of the kidney clock may affect the progression of diabetic nephropathy by stimulating renal tubular gluconeogenesis and glucose reabsorption or by damaging renal cells (Solocinski et al., 2015; Nikolaeva et al., 2016; Ansermet et al., 2022). In the progression of DR, multiple abnormal circadian rhythms including circadian disruption of systemic blood pressure (impaired nocturnal blood pressure), decreased melatonin levels (peak levels and amplitude), weakened daily cycling of enzymes for fatty acid β-oxidation, increased amplitude of inflammatory markers and loss of autophagic protein circadian rhythm all play important roles (Wang et al., 2014; Mateo-Gavira et al., 2016; Vancura et al., 2016; Coughlin et al., 2017; Hassan et al., 2017; Lemmer and Oster, 2018; Qi et al., 2020). However, the role of GCK in this process remains unclear. Current research has found that diabetic nephropathy is associated with the rs780094 polymorphism in the glucokinase regulatory protein (GCKR) gene, but the specific molecular mechanisms remain to be elucidated (Liu and Wan, 2023).

## 6 Targeting the circadian rhythm for the treatment of diabetes and its complication

**FIGURE 3:**
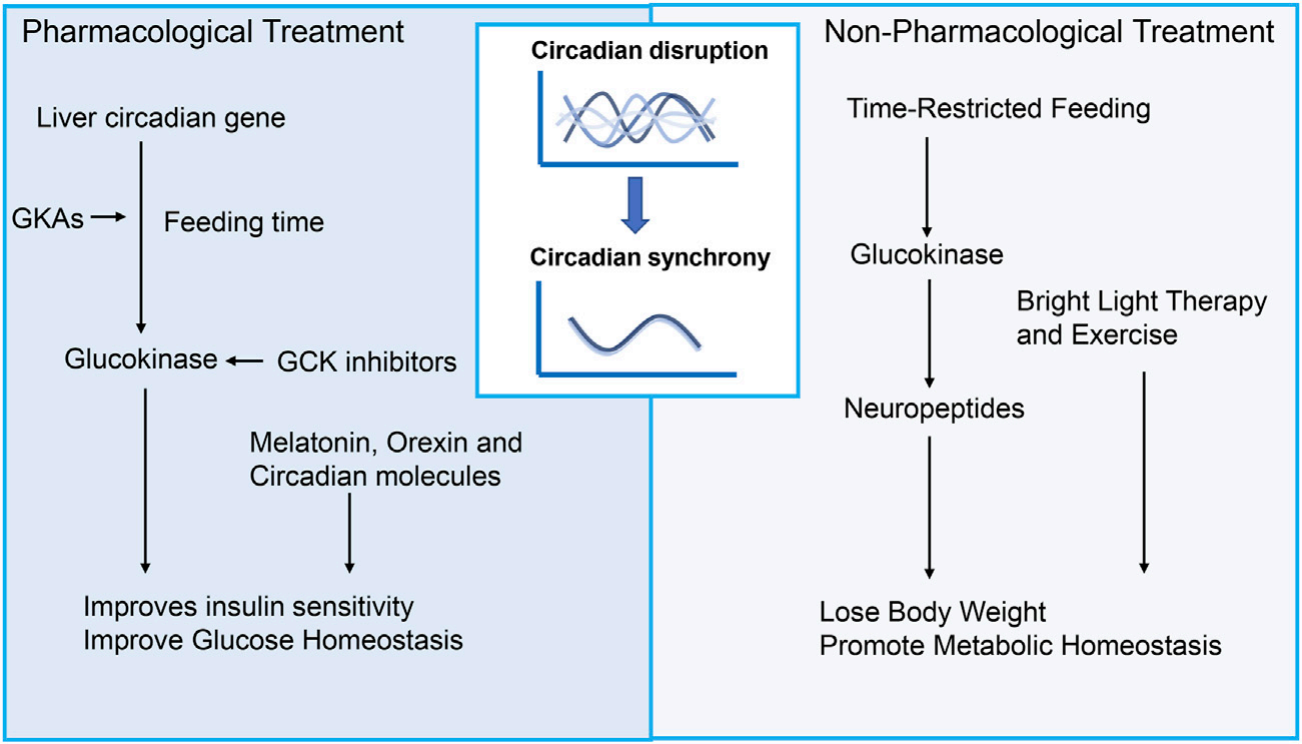
Treatment for diabetic complications targeting circadian rhythms. Promising therapeutic strategies targeting circadian disruption for diabetes and its complications can be divided into pharmacological treatment and non-pharmacological treatment. Pharmacological interventions targeting feeding-fasting and sleep-wake disorders encompass drugs that act upon GCK signaling, melatonin, orexin, and circadian molecules. In addition to these approaches, non-pharmacological treatments involving environmental and lifestyle adjustments hold promise for managing diabetic complications. Such treatments may include light therapy, time-restricted feeding, sleep intervention, scheduled activity, and combinations thereof. GCK, Glucokinase; GKAs, Glucokinase activators.

Currently, there are various treatments for the circadian rhythm of diseases. The main treatment methods can be divided into pharmacological treatment and non-pharmacological treatment (Figure 3). The first is pharmacological interventions for feeding-fasting and sleep-wake disorders. Besides, drugs that directly manipulate the core oscillator is a new perspective to circadian medicine. Non-pharmacological treatment of environmental and lifestyle regimens can also restore circadian rhythm, including light therapy, time-restricted feeding, sleep intervention, scheduled activity and combination.

### 6.1 Non-pharmacological treatments

Various lifestyle changes, such as timed meals, sleep, and physical activity, have been studied for their effectiveness in restoring circadian rhythms and treating glucose disorders. Recent years have seen remarkable progress when it comes to feeding habits, such as Time-restricted feeding (TRF), which involves granting access to food for 8–9 h during the active phase, and has been demonstrated to be very effective in preventing and treating metabolic disorders, particularly diabetes. Studies have found that TRF improves glucose tolerance and insulin resistance, reduces lipid accumulation, and influences the SCN by regulating the expression of GCK and neuropeptides such as NPY and AgRP in hypothalamus and hippocampus (Chaix et al., 2014; Tacad et al., 2022). Furthermore, TRF has significant effects on peripheral organs such as the liver and pancreas. In the pancreas, improves β-cell function through the autophagy-lysosomal pathway, reducing β-cell apoptosis, and increasing insulin sensitivity (Liu et al., 2017; Marinho et al., 2020). In the liver, TRF improves glucose and lipid metabolism by restoring circadian rhythms of core clock gene, reducing gluconeogenesis *via* CREB phosphorylation, and enhancing glucose utilization through glycogen synthesis and the pentose phosphate pathway (Chaix et al., 2019). However, the involvement of GCK in the effects of TRF on peripheral tissues remains to be elucidated. In addition, TRF has shown promising protective effects on various diabetic complications, such as nephropathy, retinopathy, neuropathy and cardiovascular diseases (Beli et al., 2018; Malinowski et al., 2019; Yang et al., 2022). Other circadian rhythm-related therapies, including light therapy and exercise, have also been shown to effectively improve sleep, reduce insulin resistance, and lower glycated hemoglobin levels (Boulé et al., 2001; Colberg et al., 2016; Wang et al., 2024).

### 6.2 Pharmacological treatment

#### 6.2.1 Glucokinase activators (GKAs) and glucokinase inhibitors

Activating GCK is a promising strategy for reducing glucose levels. Under hyperglycemia, GKAs promote insulin secretion and glycogen synthesis, while during fasting, GCK supports glycogenolysis and gluconeogenesis for glucose supply. However, GKAs can cause side effects like hyperlipidemia and hepatic steatosis. Early studies indicated that while GKAs lower glucose, they may increase liver lipids and inflammation. Tobias Kroon et al. found that timing GCK activation to feeding time improves insulin sensitivity, reverses liver steatosis, and reduces fibrosis markers (Kroon et al., 2022). This may be related to the circadian rhythm of hepatic glucose metabolism. As previously mentioned, under physiological conditions, during feeding time, the circadian rhythm genes in the liver regulate GCK to lower blood glucose levels. Taking GKAs during feeding aligns with this rhythm, which may help minimize side effects. The novel GKA, dorzagliatin, taken before meals, effectively lowers glucose without severe hypoglycemia, though slight increases in TG and TC were observed, posing potential risks (Jiang et al., 2024).

Recent study suggests that hyperactivation of glucose metabolism, leads to β cell function decline in diabetes (Brock et al., 2002). GCK plays an important role in glucose metabolism. Therefore, reducing GCK levels to lower glucose metabolism to normal levels may be a useful alternative strategy for protecting β-cells function. Existing study has found that inhibiting GCK levels *in vivo* or *in vitro* can maintain β-cell function and quality. However, the inhibition of GCK must consider whether it may lead to the occurrence of hyperglycemia. A special subtype of diabetes, glucokinase-maturity onset diabetes of the young (GCK-MODY), is characterized by patients who may have mild hyperglycemia, but their blood sugar levels do not further increase without medication, and the incidence of diabetes complications does not increase. In terms of side effects, GCK inhibition does not lead to hypoglycemia or abnormal blood lipid levels (Ashcroft et al., 2023). However, as previously mentioned, GCK inhibition seems to increase the risk of diabetic neuropathy and atherosclerosis. Further research is needed to determine whether combining GCK inhibitors with circadian rhythm can bring better therapeutic outcomes.

#### 6.2.2 Other pharmacological treatment

Other circadian rhythm-related medications include melatonin, orexin, and circadian molecules. Melatonin is a natural neurohormone synthesized from tryptophan, produced by the pineal gland and keeps the circadian rhythm (Arendt and Skene, 2005). Melatonin has been demonstrated to entrain the circadian system, and has been found to possess sleep-promoting properties, improve glucose homeostasis, and reduce insulin resistance (Gooley et al., 2011; Forrestel et al., 2017; Vasey et al., 2021). Orexin is a neuropeptide from the lateral hypothalamus (LH) crucial for regulating sleep and feeding behaviors (Tsujino and Sakurai, 2009). Furthermore, it has been reported that CRY stabilizers, REV-ERB agonists, and ROR agonists - which target the molecular clock directly - show promise in improving obesity and glucose metabolism in diabetic animal models (Solt et al., 2012; He et al., 2016; Humphries et al., 2016). Research on the relationship between these drugs and GCK remains limited. In terms of complications of diabetes, these medications help improve diabetic neuropathy, retinopathy, and macrovascular complications (Tsuneki et al., 2015; Niknia et al., 2019; Patel et al., 2022).

## 7 Conclusion

Diabetes mellitus is a globally prevalent metabolic disorder with increasing incidence and chronic complications due to long-term hyperglycemia. Disruptions in the body’s 24-h circadian rhythm are increasingly linked to severe mental and physical health impacts. GCK, which is expressed in multiple organs in the human, may play an indispensable role in these processes. In this review, we analyzed the effect of circadian rhythm disorder on diabetic complications, explored the potential molecular mechanism of circadian rhythm involvement in diabetic complications, with a particular focus on the role of GCK, and discussed the research regarding the treatment of diabetic complications through circadian rhythm.

GCK, as a key target for circadian rhythm intervention, has inconsistent effects on glucose or lipid metabolism in different tissues and under different conditions. Therefore, it is important to develop appropriate GCK-targeted intervention strategies based on the specific circadian disruptions in different tissues of diabetic patients. Most current research on circadian rhythms and pharmacological effects relies on *in vitro* or animal models, lacking substantial clinical data. These relationships highlight the need for further research to understand how circadian rhythms interact with external factors and disease processes for better prevention and treatment of diabetes and its complications.
